# *In-situ* freeze-drying - forming amorphous solids directly within capsules: An investigation of dissolution enhancement for a poorly soluble drug

**DOI:** 10.1038/s41598-017-02676-2

**Published:** 2017-06-06

**Authors:** Abdulmalik Alqurshi, K. L. Andrew. Chan, Paul G. Royall

**Affiliations:** 1 Department of Pharmaceutics and Pharmaceutical Technology, College of Pharmacy, Taibah University, Almadinah, Almunawarah Kingdom of Saudi Arabia; 2 King’s College London, Institute of Pharmaceutical Science, Franklin-Wilkins Building, 150 Stamford Street, London, SE1 9NH UK

## Abstract

Conversion into the amorphous form enhances the dissolution of poorly soluble drugs, however the barrier to market for medicines containing an amorphous drug is poor stability. The aim was to produce the amorphous form of a drug within a capsule, without thermal or mechanical stress during manufacture. To facilitate this aim, the mechanism for drug-polymer interaction was explored. Nifedipine and polyvinylpyrrolidone were dissolved in tert-butanol at different drug/polymer ratios. These solutions were dispensed into gelatin capsules and freeze-dried. Differential scanning calorimetry (DSC) & novel FT-IR analysis based on peak symmetry measurements confirmed the absence of crystallinity when polyvinylpyrrolidone exceeded 50%w/w. Capsules containing 10 mg of nifedipine were amorphous and stable for over 3 months at ≈40 °C. Evidence of hydrogen bonding between the N-H group of nifedipine and the C=O group of PVP was observed and this interaction inhibited nifedipine crystallisation. PVP’s high affinity for water and the nifedipine-polymer interaction lead to a significant dissolution rate enhancement. The freeze-dried capsule, 10%w/w nifedipine/PVP, had the highest dissolution rate constant of 0.37 ± 0.05 min^−1^, and the lowest time to achieve 50% dissolution or t_1/2_ of 1.88 ± 0.05 min. This formulation reached 80% dissolved in less than 6 min whereas the equivalent marketed liquid filled nifedipine capsule took 3 times longer to reach 80% dissolution.

## Introduction

The biopharmaceutical classification system (BCS) ranks drugs in accordance to their solubility and permeability^1^, the dominating factors that influence the oral bioavailability of drugs^2, 3^. Class II BCS, drugs with high permeability but low solubility, form a large number of the lead compounds generated by pharmaceutical research, but their low oral bioavailability reduces class II’s developability into medicines^4^. Due to limited aqueous solubility, the absorption of class II BCS drugs is hindered^1^. This may be improved through formulation strategies, for example rendering the drug into its amorphous form^3, 5^.

An effective approach for enhancing the kinetic solubility and dissolution rate is to molecularly disperse a drug in a solubilizing carrier, thus inhibiting crystallisation and maintaining the drug within a disordered or amorphous structure^6^. Despite the benefits of this strategy, amorphous drugs are not widely used in the pharmaceutical industry^6, 7^ as most methods of generating amorphous materials expose the drug to thermal or mechanical stress, which leads to chemical degradation and re-crystallisation of the amorphous solid. For example, solid polymer-drug solutions maybe prepared using hot-melt extrusion (HME), a process limited to thermally stable drugs^4, 8^. Thus, less aggressive methods for rendering poorly water soluble drugs (PWSD’s) into amorphous medicines are required^6^. Solvent evaporation methods^9^, where a drug and polymer are dissolved in a common volatile solvent, which is then removed via spray or freeze-drying ensures moderate to low heat exposure^10–17^.

Water is the principle solvent used for freeze-drying^18^ which puts PWSD’s at a disadvantage, as acceptable concentrations of the PWSD in the aqueous feed solutions are impossible to achieve. Some organic solvents may be used in combination with, or in place of, water, given they have a high freezing point, −10 °C to 30 °C, and a relatively high vapour pressure below their freezing point, >0.26mbar^19, 20^. Tert-butanol (TBA), with a freezing point of 24 °C and a vapour pressure of 35.72 mbar at −20 °C, is a potential organic freeze-drying solvent^21–25^.

The dissolution of freeze-dried products is rapid, due to the large surface area created by their porosity and low-density. However secondary processing of these highly porous materials is impossible and typically freeze-dried samples are re-hydrated and delivered via an injection. Filling conventional capsules with delicate freeze-dried cakes^8^ to form an oral dosage form is impractical, because the samples will not flow easily due to their low density. Compressing freeze-dried cakes into conventional tablets with desirable hardness, would remove their much needed porosity and open structure. More importantly, the physical and thermal stress of tableting may induce re-crystallisation of the PWSD, taking away the dissolution benefits of the amorphous form. A successful alternative to maintaining the pre-request of an amorphous structure is the freeze-dried delivery platform Zydis®, where the tablet is produced with a predominantly crystalline structure. However formulating a PWSD into an oral-dispersible Zydis® tablet creates issues around taste, as PWSDs are known for their bitter flavour^26^, which can significantly reduce patient compliance. Zydis® tablets are designed to disintegrate in very small amounts of saliva prior to swallowing, this can lead to drug precipitation prior to reaching the absorption site^27^. A novel approach is to freeze dry solutions *in-situ* within a physically protective and easy to handle vessel. Such vessels must allow the intact freeze-dried cake to be protected from any potential physical damage that may come with packaging and handling. Conventional hard gelatin capsule shells are the perfect vessel to use, assuming they withstand liquid loading and freeze-drying.

Our study assessed the utilization of *in-situ* freeze-drying within capsules for the delivery of amorphous drugs in their solid form via the oral route. This novel technology sought to enhance the dissolution of PWSDs and nifedipine was used to test this approach. Nifedipine, a calcium channel blocker used in the treatment of cardiovascular disorders and a class II BCS PWSD model^28–31^. Interestingly for a marketed drug, nifedipine has poor chemical stability as it is particularly susceptible to light induced photo degradation^31^. Nifedipine is practically insoluble in water e.g. 0.0056 g/L at pH7^31^ and when rendered amorphous it has a low glass transition temperature (*T*_*g*_) of 46.2 ± 0.2 °C^32^. To prevent re-crystallisation, amorphous drugs must be stored at least 50 °C below their *T*_*g*_^33, 34^, thus amorphous nifedipine is unable to remain amorphous if stored at room temperature.

Molecularly dispersing nifedipine in a large molecular weight, intrinsically amorphous carrier, is a successful approach to form a solid material with a single *T*_*g*_, > 46 °C^6, 35^. Polyvinylpyrrolidone (PVP) is one of the most successful carriers due to its propensity for forming solid solutions with drugs^6^. The resulting mixtures have higher glass transition temperatures with respect to the pure drug and therefore prevent their re-crystallisation^36, 37^.

The aim of the research reported in this paper was to develop a novel *in-situ* freeze-dried capsule presenting a poorly soluble drug in its amorphous form that shows enhanced drug dissolution and stability. To facilitate this aim, a mechanistic investigation of the interaction of nifedipine with PVP was conducted.

## Results and Discussion

### Method development

**Figure 1:**
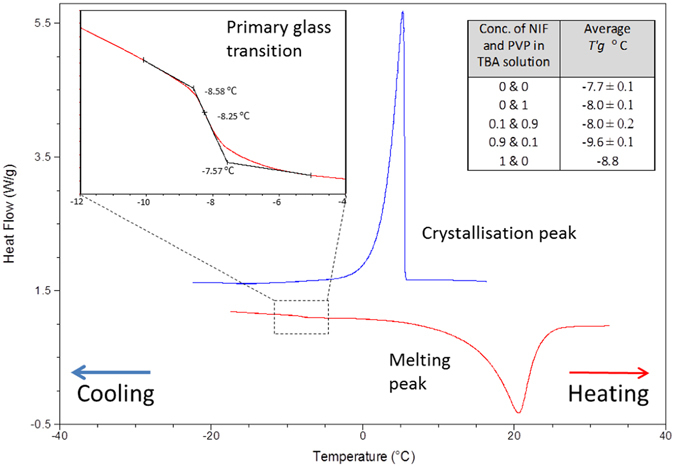
Thermogram of a liquid sample of NIF and PVP (with a target w/w percentage of 10% NIF in PVP) dissolved in TBA The sample was cooled to temperatures below −20 °C and heated at a rate of 10 °C/min. *T’*_*g*_ determined to be −8.3 °C. Table in top right corner presents average *T’*_*g*_ values of feed solutions containing highest and lowest concentrations, in % w/v, standard deviation, ±, is shown for n = 3.

The collapse temperatures of the freeze concentrated solutions (*T*_*c*_) were predicted from the primary glass transition temperatures (*T*_*g*_ *’*) of the equivalent solutions of nifedipine and PVP in TBA. *T*_*g*_ *’* ranged between −9.6 ± 0.1 and −7.7 ± 0.1 °C (n = 3), Fig. 1. The effect of nifedipine and PVP concentrations on the *T*_*g*_ *’* of the freeze concentrated solutions was not significant. *T*_*c*_ is usually 2 °C higher than the *T*_*g*_ *’* ^18^, therefore *T*_*c*_ was predicted to lie between −7 and −5 °C. Thus collapse was avoided within the capsules by maintaining a product temperature (*T*_*p*_) 5 °C below the lowest *T*_*c*_ during the primary drying cycle. Once freeze-drying was complete, the residual solvent per capsule was well below the limit of human consumption stated by the National Sanitation Foundation (NSF) of 1.0 mg/kg-day^38^. Thus for the 10% w/w nifedipine (NIF) in PVP formulation and based on a 75 kg patient, a maximum of 6 capsules per day may be consumed following NSF guidelines^38^. Other organic solvents that can enable the dissolution of NIF and PVP, such as ethanol and DMSO, may be used in formulating amorphous nifedipine in PVP, however the physicochemical properties of TBA, for example its vapour pressure of 35.72 mbar below its freezing point, allows TBA to be removed during a freeze drying cycle. Furthermore, the use of TBA as a solvent for freeze-drying pharmaceutical products is currently in limited practice, CAVERJECT® is one of such marketed formulation^21^.

### Quality control

**Figure 2:**
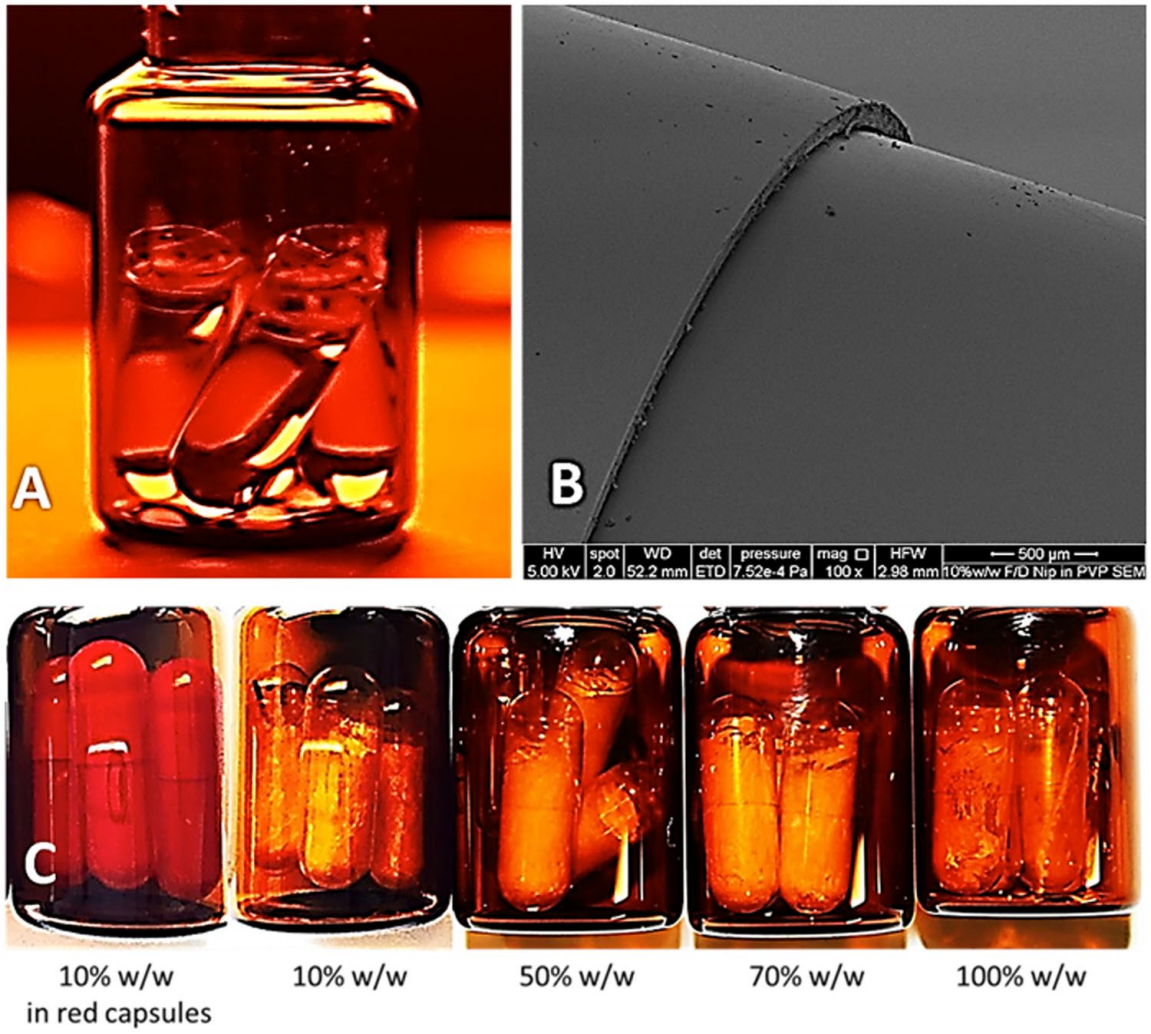
(**A**) Clear gelatin capsules filled with 0.5 mL of liquid TBA (40 ± 0.5 °C). (**B**) SEM of *in-situ* red capsule freeze-dried formulation. The capsule bottom part was involved in the freeze-drying process, while the top part (left of the image) was not. The structure of both looks identical under SEM. (**C**) *In-situ* capsule freeze-dried samples of nifedpine in PVP. Left to right showing low to high w/w % of NIF in PVP. All formulations were designed to contain 10 mg of nifedipine whatever the amount of PVP present.

All formulations were designed to contain a constant dose of nifedipine whatever the amount of PVP present, and this 10 mg dose was equivalent to the available/marketed liquid filled immediate release capsules. Formulations with ≤70% w/w NIF in PVP showed no shrinkage or collapse, indicating successful freeze-drying, while >70% w/w NIF in PVP a loss of structural integrity was observed, Fig. 2. At such low levels of PVP, re-crystallisation of nifedipine occurs disrupting the continuous nature of the freeze-dried cake by phase separation. This was further confirmed using differential scanning calorimetry, polarised microscopy and FT-IR analysis, which are discussed in the following sections.

The effect of the freeze-drying on the quality of hard gelatin capsule shells was investigated following the World Health Organization (WHO) general monograph for quality control of hard gelatine capsules^39^. Visual inspection of hard gelatin capsules, that had been filled with pure TBA and exposed to the same freeze-drying cycle as capsules containing TBA solutions with nifedipine & PVP, (otherwise referred to here, as treated capsules), maintained a smooth and undamaged body (n = 20). Under scanning electron microscopy a capsule bottom (treated) and top (untreated) showed no topographical differences Fig. 2. Liquid TBA, 0.5 mL, was placed inside red hard gelatin capsule shells for 60 min at 40 ± 0.5 °C, Fig. 2. Physical examination of the capsule shell showed no damage imposed by liquid TBA, further, full UV absorbance spectrum, of the liquid TBA, showed no scattering or absorbance, indicating no leaching of any red dye or gelatin from the capsule into TBA (n = 3).

FT-IR spectra of treated and untreated capsules had a correlation of >97% indicating no chemical damage in the treated hard gelatin capsule shells. The capsules were also tested following the international pharmacopeia guidelines for disintegration^39^. All treated capsules (n = 6) disintegrated in under 6 min with untreated hard gelatin capsules showing a similar response. Thus treated capsule shells complied with the international pharmacopeia limits for hard gelatin capsule disintegration times of <30 min^39^. The uniformity of mass of the treated hard gelatin capsules (n = 20) had ≤2% deviation from the average weight, therefore complying with the WHO limit of 10% deviation from the average weight. Thus the integrity of the gelatine capsules was maintained throughout the freeze-drying cycle when TBA was used as a solvent.

### Uniformity of weight and drug content

The *in-situ* freeze-dried (FD) capsule 10% w/w NIF in PVP formulation complied with the British pharmacopeia (BP) limits of uniformity of weight Table 1S ^39^. Its uniformity of weight value expressed as relative standard deviation (RSD%) and that of the liquid filled, marketed formulation (TEVA 10 mg nifedipine fast release soft gel capsule) both had RSD’s below 2%. The high content of PVP, in the 10% w/w NIF in PVP formulation, compared to other FD formulations, gave its FD cake increased structural integrity and avoided losses during the freeze-drying cycle and the recapping process. This had a positive effect on the drug content (Figure 1S), where the 10% w/w NIF in PVP formulation complied with the BP limits of drug content (10 ± 0.5 mg).

### Detecting crystalline nifedipine

The extent of the amorphous dispersion of nifedipine within PVP, i.e. the absence of crystallinity, was investigated by optical microscopy, FT-IR and thermal techniques.

### Differential scanning calorimetry

**Figure 3:**
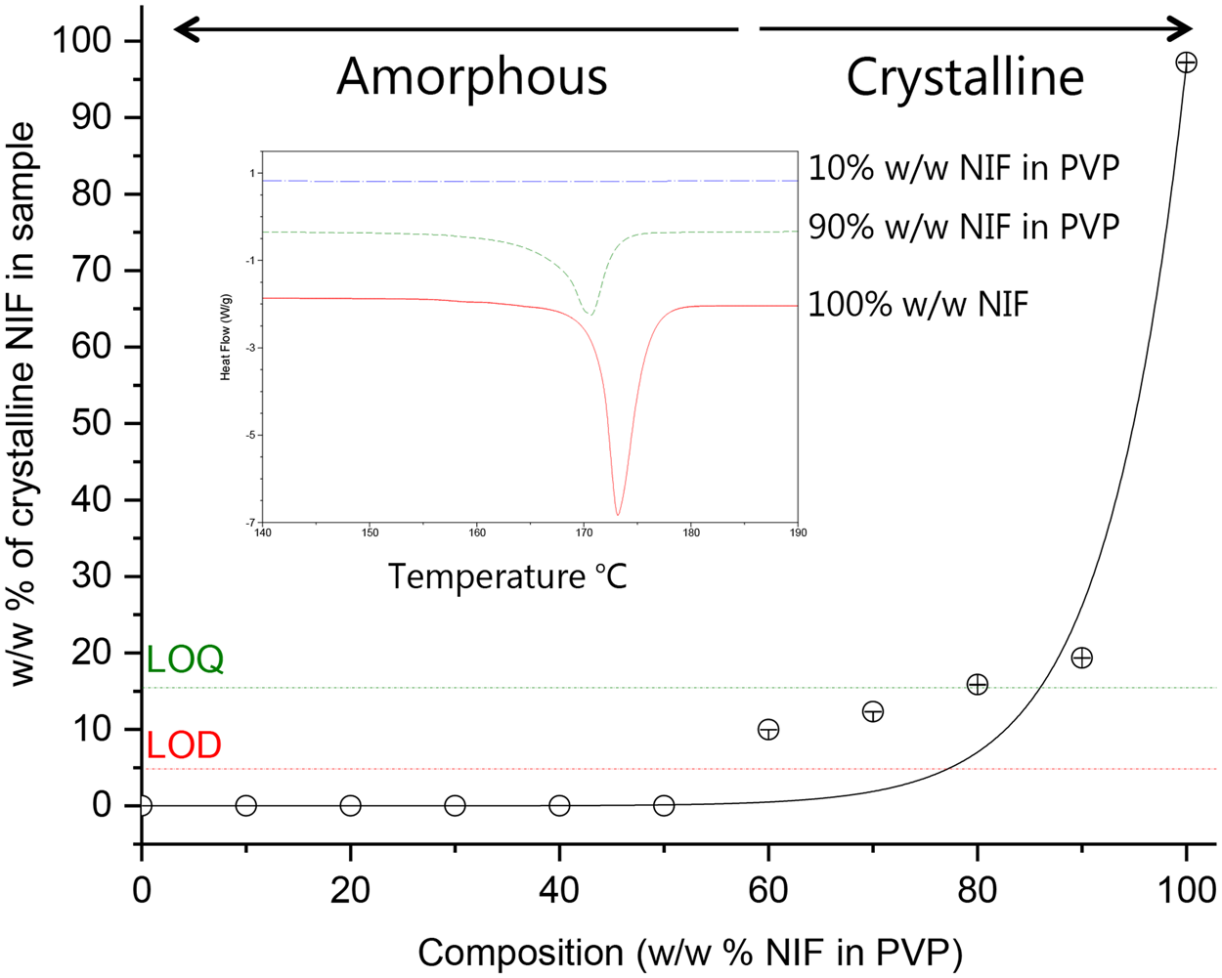
Crystallinity phase diagram for freeze-dried samples at first heat. The w/w % of crystalline NIF in samples was calculated using a calibration graph based on physical mixes of NIF in PVP. Error bars represent the standard error of n = 3. Thermogram showing first-heat of freeze-dried formulations 100, 90 and 10% w/w NIF in PVP. DSC cycle include a ramp from −10 to 190 °C at a rate of 10 °C/min.

Nifedipine as received melted at 172.4 ± 0.1 °C which falls within the literature onset range of 172–174 °C^15^, confirming the crystalline stable modification-I, the α-polymorph. The as received PVP K10 showed an average *T*_*g*_ of 146.1 ± 2.8 °C. The melting peak of nifedipine for the freeze-dried formulations broadens and diminishes in size as the w/w % of PVP increases, indicating a decrease in the crystallinity, Fig. 3. The melting peak disappears completely for the freeze-dried formulations containing ≤50% w/w NIF in PVP, which is indicative of a predominantly amorphous structure (Fig. 3).

**Figure 4:**
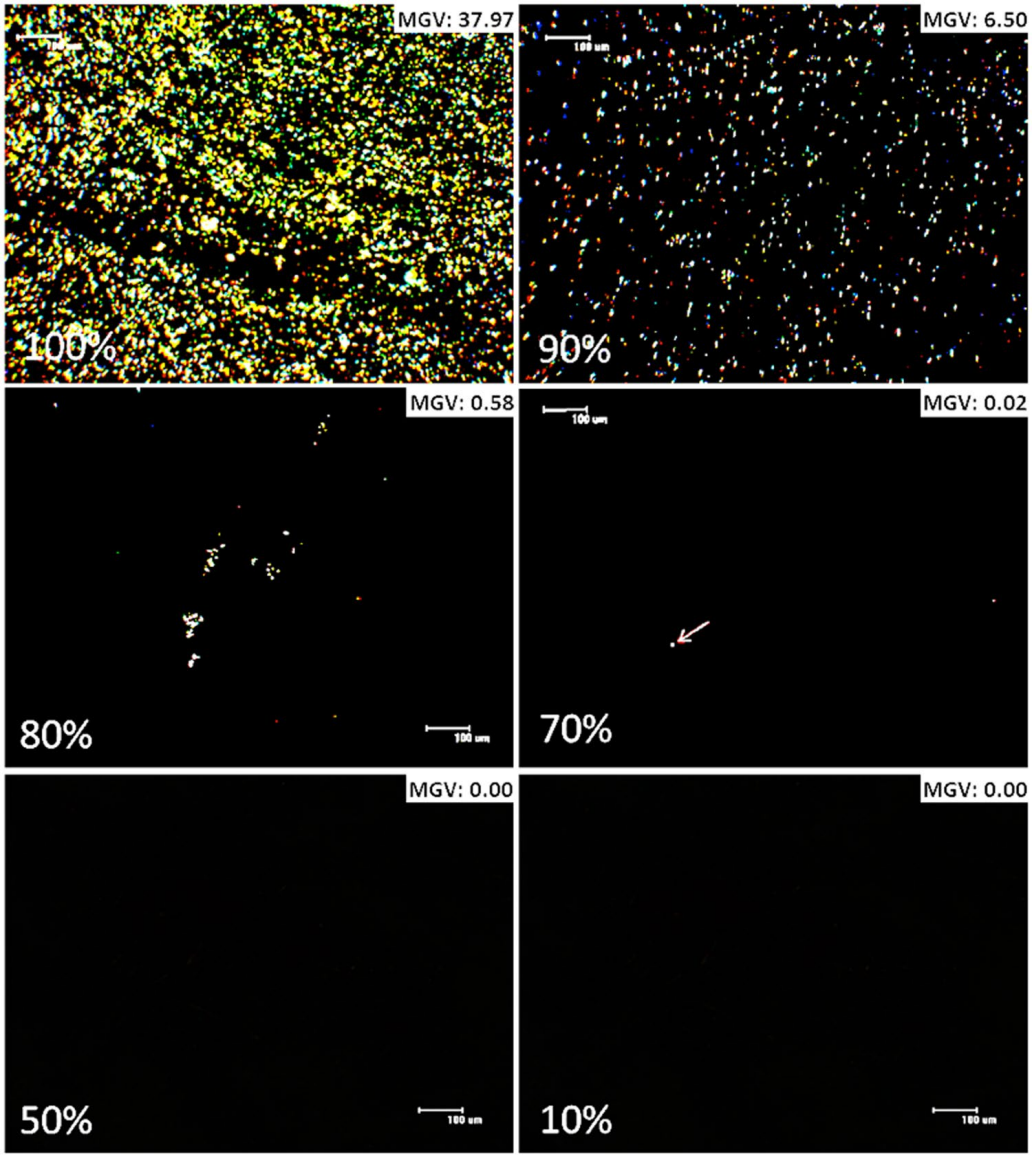
Polarized microscopy images of freeze-dried 100%, 90%, 80%, 70%, 50 and 10%w/w NIF in PVP. The crystal caused birefringence was observed to gradually decrease as the w/w% of NIF in PVP is reduced, while maintaining a constant 10 mg dose of NIF per capsule. The mean grey value (MGV) is listed on the top right corner of each image. 10 and 50% w/w NIF in PVP show no birefringence, thus MGV is at its lowest, while 90 and 100%w/w NIF in PVP show a relatively high amount of birefringence, this is shown in their shown in their MGV.

Environmental scanning electron microscopy (ESEM) images, in Figure 4S (supplementary information), showed no micro-phase separation in freeze-dried samples at or below 50% w/w NIF in PVP, thus further confirming molecular homogeneity.

For physical mixtures, the melting enthalpy peak area (PA) of crystalline NIF was linearly correlated to the content of crystalline nifedipine in samples containing NIF in PVP ($[{\Delta }_{melt}H=(1.01\times \frac{w}{w} \% \,NIF)-8.06]$, R^2^ = 0.99). This was used to determine the w/w% of crystalline nifedipine in the freeze-dried formulations^40, 41^. The limit of detection (5.1%w/w) and quantification (16.6%w/w) calculations were based on the calibration curve method described in the international conference on harmonisation of technical requirements for registration of pharmaceuticals for human use (ICH)^42^. Similar values for a range of pharmaceutical materials were determined by Buckton and Darcy^43^. Applying this equation, led to the phase diagram for nifedipine in the samples (Fig. 3). Freeze-dried formulations containing ≤50% w/w NIF in PVP were amorphous, above this crystalline nifedipine was observed. HPLC showed heat-cycled formulations contained nifedipine thermal degradation products, for example a 4 cycle DSC experiment (heat-cool-heat-cool) caused a loss of 20.9 ± 6.4%. A single cycle DSC experiment (i.e. heat only) is therefore expected to cause a loss of ≈5.2%, which is equal to the limit of detection (LOD) of the DSC assay for crystalline nifedipine (Fig. 3 and Table 2S). Thus microscopy and IR approaches, which operated at room temperature, were used to confirm the absence of crystallinity in the high PVP content formulations.

### Cross polarization microscopy (CPLM)

The anisotropic structure of crystalline nifedipine^44^, was seen by the observation birefringence when the crystals were viewed by CPLM, (Fig. 4)^45, 46^. Amorphous nifedipine, due to its disordered structure, did not show such a pattern and appeared dark under 90° orientation of the cross polarizer. CPLM confirmed that less than 50% w/w FD NIF in PVP was the cut-off point where crystalline nifedipine was not detected. Formulations with above 70% w/w NIF in PVP showed a gradual increase of crystallinity as the w/w % of nifedipine increased. To quantify the CPLM analysis, images were analysed using ImageJ software providing the mean grey value (MGV). MGV of polarized images containing no birefringence were equal to 0.00 arbitrary units (AU). The presence of crystals and therefore birefringence causes the measured MGV to increase, where for example FD 100% w/w NIF showed an MGV of 36.5 (Fig. 4).

### ATR-FT-IR

**Figure 5:**
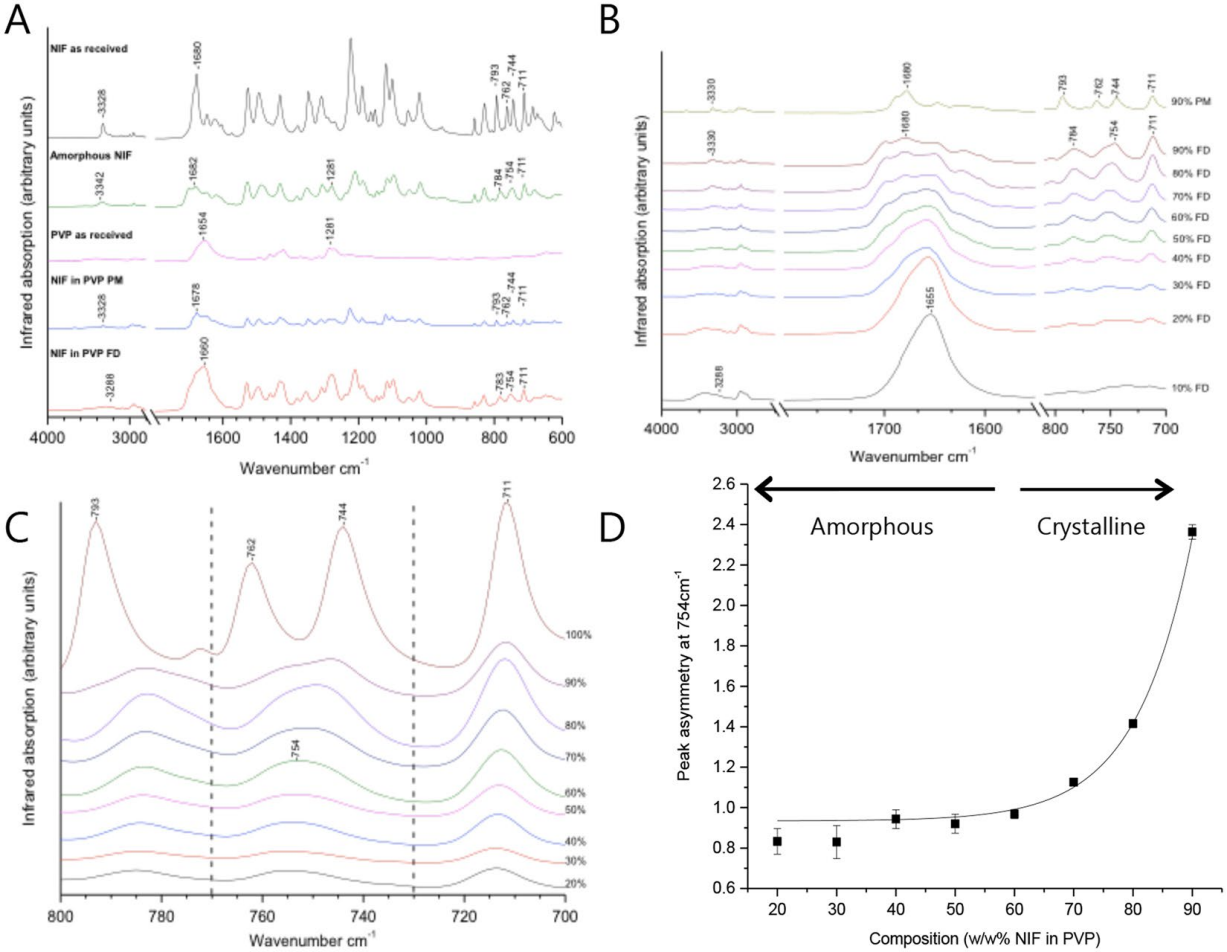
(**A**) FT-IR spectra of crystalline nifedipine as received, amorphous nifedipine (produced by heat melt), PVP as received, 50% w/w crystalline NIF in PVP as physical-mix (NIF in PVP PM) and Freeze-dried 50% w/w NIF in PVP (NIF in PVP FD). FT-IR measurements were repeated to ensure data reproducibility (n = 3). (**B**) N-H (3288–3330 cm^−1^), Carbonyl (1660–1680 cm^−1^) and out of plane δ(C-H) vibration of the ring (800–700 cm^−1^) regions of the FT-IR spectrum for the full range of freeze-dried NIF in PVP formulations compared to the 90% w/w NIF in PVP physical-mix of crystalline NIF in PVP. All percentages listed are of NIF in PVP. FT-IR measurements were repeated to ensure data reproducibility (n = 3). (**C**) FT-IR spectra (800–700 cm^−1^ only) of freeze-dried formulations 20–100% w/w NIF in PVP. Peak symmetry change at 754 cm^−1^ (peak distinctive of amorphous nifedipine) was examined. 90% w/w NIF shows a clear left shoulder. FT-IR measurements were repeated to ensure data reproducibility (n = 3). Spectra presented in (**A**), (**B**) and (**C**) where corrected for baseline using the software PerkinElmer spectrum 10. Additionally spectra presented in (**B**) & (**C**) had a PVP spectrum subtraction. (**D**) Monitoring NIF crystallinity in freeze-dried formulations using FT-IR peak symmetry at 755 cm^−1^. A change in peak symmetry is indicative of a change in physical state, as amorphous NIF presents a single broad and asymmetric peak at 754 cm^−1^ and crystalline NIF presents 2 separate peaks at 762 cm^–1^ and 744 cm^−1^, the gradual change from the broad amorphous peak to the double crystalline peaks can be quantitatively measured through peak symmetry measurements. Error bars represent standard error of n = 3.

ATR-FT-IR spectrum of nifedipine as received had the main characteristics of the thermally stable α-nifedipine spectrum^30^, which exhibits relatively low C = O (doublet centred at 1680 cm^−1^) and N-H (3327 cm^−1^) wavenumbers, compared to amorphous nifedipine (Fig. 5). This is due to strong intermolecular hydrogen bond interactions between the ester carbonyl and the NH group of the adjacent nifedipine molecule^30^. The FT-IR spectrum of amorphous nifedipine shows a distinct difference to the crystalline nifedipine in the sharpness and definition of peaks, specifically in the 1700 cm^−1^ region^47^. The (N-H) band was also shifted to a higher wavenumber indicating less or weaker intermolecular hydrogen bond interactions in amorphous nifedipine.

Freeze-dried nifedipine in PVP (Fig. 5A) also shows clear distinctive IR features from the crystalline physical-mix nifedipine in PVP. However the 1678 cm^−1^ band is thought to be affected by the water band bending mode absorption at ≈1640 cm^−1^ ^29^. Additionally, PVP was shown to absorb in the (N-H) and carbonyl regions (Fig. 5A), which made these regions difficult to quantify crystalline nifedipine in freeze-dried formulations. The shape and location of peaks in these regions (N-H and C = O) may be used to qualitatively analyse the physical state of nifedipine in freeze-dried formulations^30^. Figure 5B presents the N-H and carbonyl regions of the full range of freeze-dried formulations. A clear change in the shape and location of the C = O peak (1660 – 1680 cm^−1^) was observed at 70 and 80% w/w NIF in PVP. These changes are due to the variance in the NIF:PVP ratio rather than an increase in crystalline nifedipine in the formulation. An alternative region that is less influenced by the change in the NIF:PVP ratio which can be used to distinctively differentiate between molecularly dispersed amorphous nifedipine in PVP and the crystalline nifedipine in PVP physical-mix is the 800–700 cm^−1^ out of plane δ(C-H) vibration of the ring regions, where PVP has very little absorbance effect (Fig. 5A and B). Pure crystalline nifedipine shows distinctive peaks at 793, 762 and 744 cm^−1^ (Fig. 5B) which are also shown in crystalline nifedipine in PVP physical-mix^29, 30^. Pure amorphous nifedipine, on the other hand, shows distinctive peaks at 784 and 754 cm^−1^ which are also observed in freeze-dried nifedipine in PVP (Fig. 5A and B). The peak located at 711 cm^−1^ is a common peak present in all nifedipine-containing spectra, independent of the physical state of nifedipine, and may therefore be used as an internal standard for quantitative analysis. Iqbal and Chan^29^ attempted to use the ratio of the crystalline indicative 762 cm^−1^ PA against the standard PA at 711 cm^−1^ to semi-quantify crystalline nifedipine in molecularly dispersed nifedipine formulations (PA = peak area). The absence of the 762 cm^−1^ peak indicated the absence of crystalline nifedipine on the outer surface of the sample, and thus samples showing PA ratios ≤0.05 where considered to be amorphous^29^.

Unlike the study performed by Iqbal and Chan^29^, this study has shown that all freeze-dried formulations do not show a peak at 762 cm^−1^ (Fig. 5B). However, a clear change in the 754 cm^−1^ peak shape accompanied with a slight change in peak position was observed (Fig. 5C). While amorphous nifedipine shows a single broad peak at 754 cm^−1^, crystalline nifedipine shows two peaks, 744 cm^−1^ and 762 cm^−1^. The gradual change from amorphous to crystalline NIF, causes the amorphous 754 cm^−1^ peak to gradually change in shape where for example, freeze-dried 90% w/w NIF in PVP shows a clear shoulder towards the 762 cm^−1^ crystalline peak, which maybe early signs of nifedipine re-crystallization, such as the formation of nifedipine dimers in small clusters.

The peak asymmetry was calculated from B/A at 10% peak height where B and A are the left and right half widths of the peak respectively. Peak asymmetry was plotted for the composition range of the freeze-dried formulations (Fig. 5D). Freeze-dried 10% w/w NIF in PVP was not included, as peaks in the range 800–700 cm^−1^ were too small for integration. Fig. 5D shows the 754 cm^−1^ peak asymmetry of ≤70% freeze-dried NIF in PVP formulations to equal 1 ± 0.2, which is considered asymmetrically acceptable by HPLC ICH guidelines^48^. Changing the composition of the freeze-dried formulation to contain >70% w/w NIF in PVP resulted in a sharp increase in the peak asymmetry, indicative of some nifedipine crystal nucleation such as clusters of dimers. This is further supported by CPLM images presented in Fig. 4, where small amount of crystals of nifedipine are observed in 70% w/w FD formulations compared to the 100% w/w formulation^46^.

### Drug Polymer interaction

**Figure 6:**
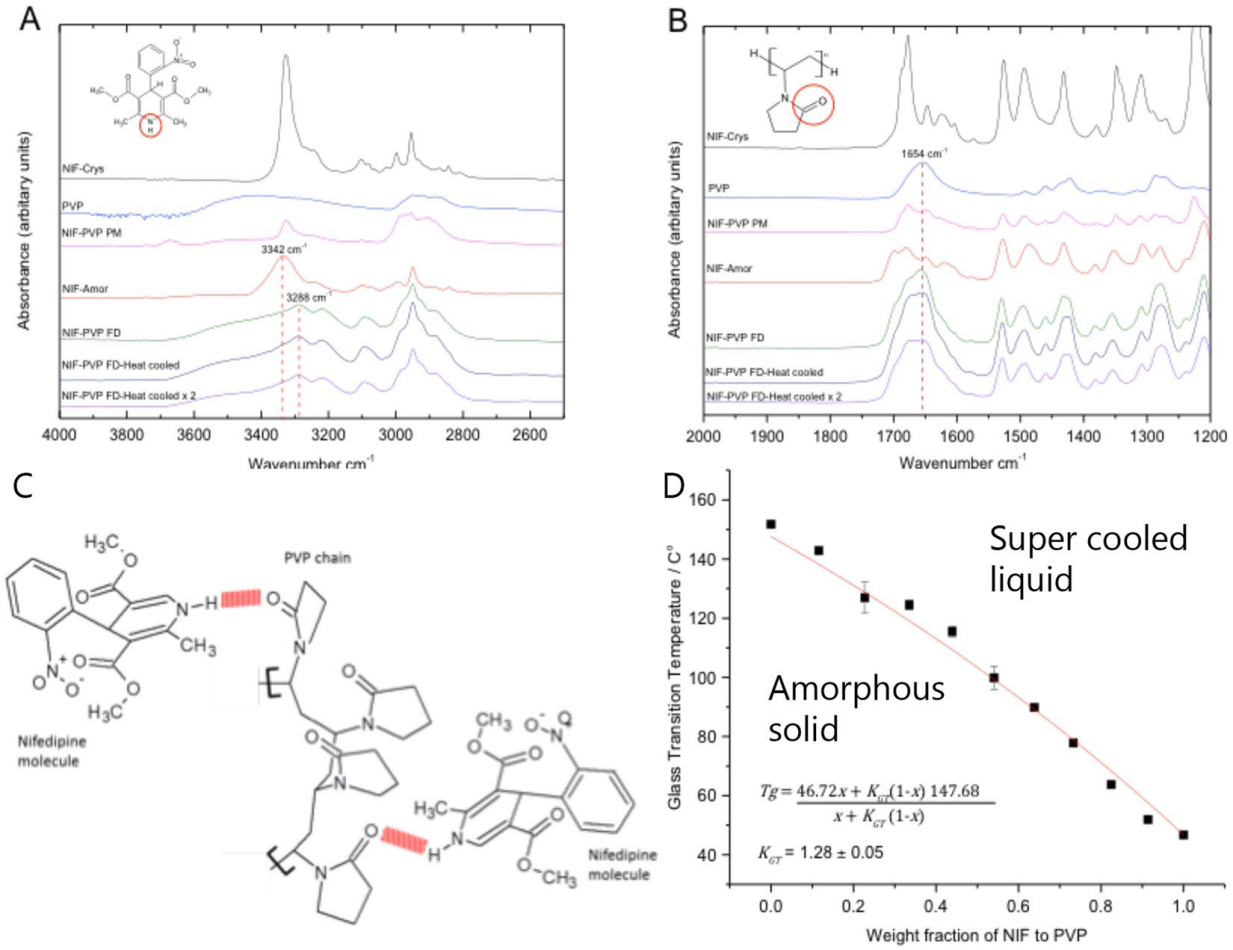
(**A**) FT-IR results show the N-H peak in pure amorphous nifedipine 3342 cm^−1^ shifts to 3288 cm^−1^ in FD formulations of nifedipine in PVP; thus indicating strong intermolecular hydrogen bonding between the N-H group of nifedipine and the carbonyl group in PVP^37, 49^. **(B)** Showing the (C = O) carbonyl group of PVP maintained at 1654 cm^−1^ in pure PVP and FD formulations: indicating that the C = O peak of PVP was not greatly influenced by the hydrogen bonding between NIF and PVP^37, 49^. FT-IR spectra presented in (A) and (B) were corrected for baseline using the software PerkinElmer spectrum 10. **(C)** A proposed model for the intermolecular hydrogen bonding between nifedipine and PVP based on the IR spectra. Hydrogen bonds are presented in red. This figure was constructed using ACD/ChemSketch. **(D)** Average *T*_*g*_ of heat cycled samples of nifedipine in PVP. The data was fitted to Gordon Taylor equation, using OriginPro, where *x* is the weight fraction of nifedipine. Error bars represent standard error of n = 3.

FT-IR results show the N-H peak in pure amorphous nifedipine 3342 cm^−1^ ^37^ shifts to 3288 cm^−1^ in FD formulations of nifedipine in PVP (Fig. 6A and B), indicating hydrogen bonding between the N-H group of nifedipine and the carbonyl group in PVP^49^ (Fig. 6C). The carbonyl group in pure PVP (1654 cm^−1^) was not seen to change in amorphous freeze-dried formulations of NIF in PVP (Fig. 6B), contradicting the observations seen in the N-H region. This was caused by the relatively low ratio of nifedipine molecules to vinyl pyrrolidone monomers, which was ≈1:25 in FD formulations of 10% NIF in PVP. As a result, the effect of hydrogen bonding on the overall C=O absorption, in PVP, is insignificant. Authors in the field, e.g. Rumondor *et al*.^49^ have made the mistake of assuming the C=O absorption at ≈1660 cm^−1^ results from hydrogen bonding between PVP and nifedipine, despite pure PVP showing the same absorption peak (Fig. 6B).

Fitting *T*_*g*_ values to the Gordon-Taylor equation is an accepted approach for characterising the interaction of the species within an amorphous dispersion^50–52^. In fact the *T*_*g*_’s for all the NIF:PVP ratios, fitted the Gordon Taylor equation (Fig. 6D), and as the equation is based on volume additivity it assumes minimal interaction between the species involved^35, 50^. The hydrogen bonding interaction between nifedipine and PVP makes little impact on the Gordon-Taylor fit for two reasons: Firstly; the weight fraction of polymer is used to generate Gordon-Taylor plots, i.e. the number of monomer units, outweighs the amount of drug present and so the interaction represented by the N-H peak shift will not greatly influence the mobility of PVP. Secondly; DSC caused chemical degradation within nifedipine during each heating cycle, the products of which will have an unpredictable effect on *T*_*g*_. However, the Gordon-Taylor plot does show the stabilising effect of PVP, by raising the *T*_*g*_ many 10 of degrees above room temperature, reducing the likelihood of re-crystallisation within the freeze-dried samples.

### Dissolution studies

Drug quantification was performed through stability indicating HPLC-UV assay, which was validated following ICH^42^ guidelines^28, 53^. This assay minimised the impact of photo degradation, however previous work^54^ indicates that degradation of nifedipine, (whether dispersed in PVP or in its as received form), during dissolution in the BP monograph recommended media is very difficult to eliminate^31^ and thus degradation was detected when dissolution experiments were extended to over 60 min.

**Figure 7:**
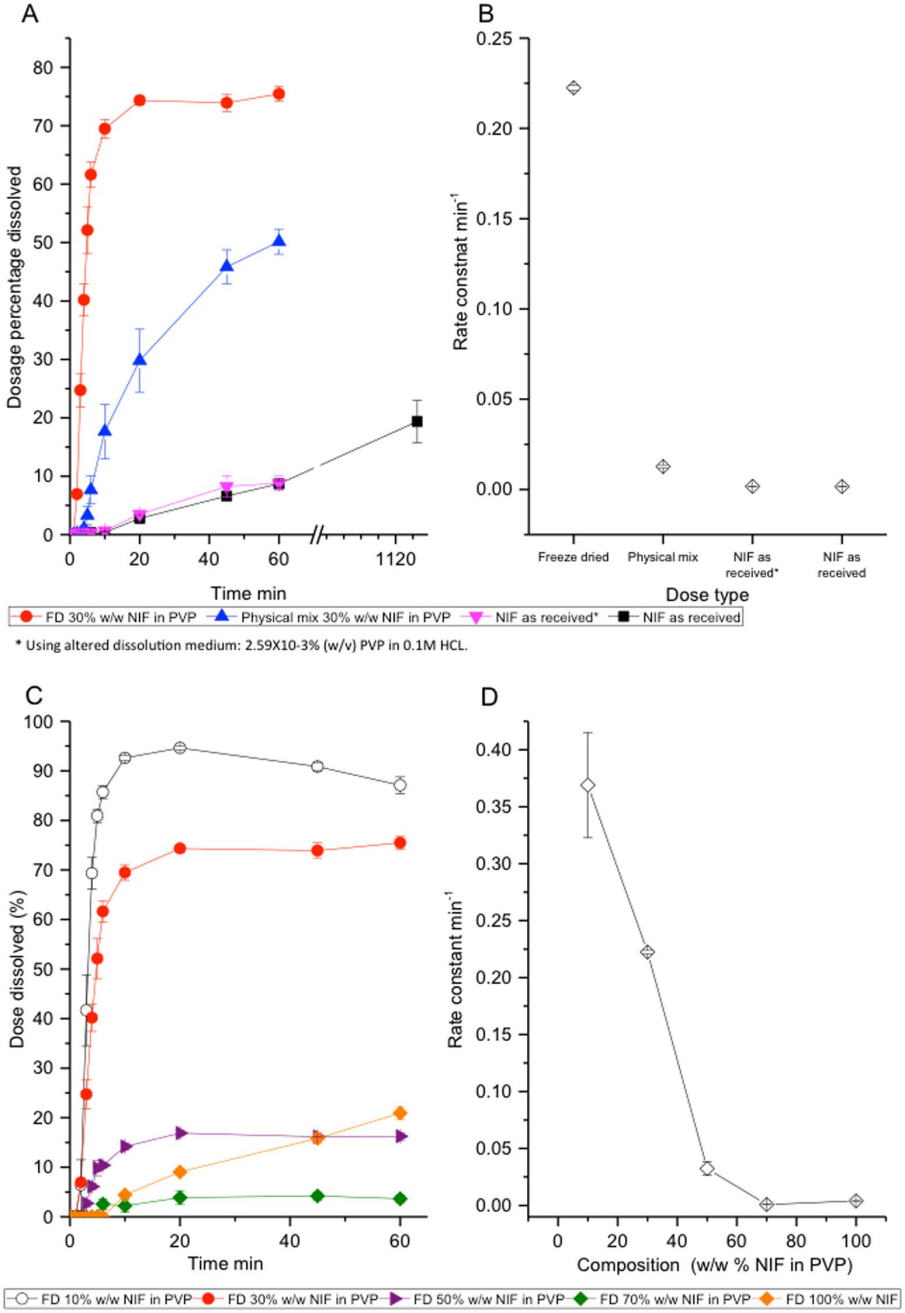
Comparing (**A**) dissolution profile (**B**) rate constant of nifedipine in different dosage types. The dissolution medium is 0.1 M HCl unless otherwise stated. Temperature of dissolution medium is 37.0 ± 0.5 °C and dissolution volume 900 mL. Test was performed following USP paddle apparatus 2. Error bars represent standard error of n = 3. Comparing (**C**) dissolution profile (**D**) rate constant of *In situ* FD nifedipine capsules with varying NIF:PVP ratios The dissolution medium is 0.1 M HCL unless otherwise stated. Temperature of dissolution medium is 37.0 ± 0.5 °C and dissolution volume 900 mL. Test was performed following USP paddle apparatus 2. Error bars represent standard error of n = 3.

Dissolution of crystalline nifedipine as received (Fig. 7A) was less than 10% of the dose after 60 min, with a *t*_*1/2*_ greater than 350 min. The addition of PVP to the dissolution medium, in concentrations equivalent to the amount of PVP in 30% w/w NIF in PVP, did not show a significant difference on *k* (*p* > 0.05), indicating that the presence of PVP in dissolution medium does not improve the dissolution of NIF. Whereas grinding PVP with nifedipine, to produce physically mixed formulations, improved *k* by approximately 20 times, showing a *t*_*1/2*_ below 35 min. This was due to the dual effect of decreasing the particle size of nifedipine and increasing the surface area, thus enhancing the rate of dissolution^55^, and increasing the surface area between the drug and polymer, and therefore allowing more efficient wetting. This effect was also observed by Broman *et al*.^56^, where enhancing the contact between polymer and drug was key for enhancing the wetting of drug, therefore providing access of the dissolution medium to the drug surface and preventing aggregation of particles. Thus dissolution of the physically mixed formulations increased the concentration of PVP in the vicinity of nifedipine particles and led to better wetting of nifedipine.

Presenting nifedipine in a freeze-dried formulation improved *k* by another 10 times (Fig. 7B), with a *t*_*1/2*_ below 4 min (Fig. 7C). Faster dissolution rates were a consequence of converting nifedipine into its amorphous form. A sharp increase in dissolution rates for freeze-dried formulations as the % w/w of NIF in PVP decreases, and thus a lowering and eventual removal of crystalline content, was seen. Similar patterns were observed for ibuprofen & albendazole in PVP solid solutions^57, 58^.

Lowering the w/w% of NIF in PVP from 30 to 10% significantly increased *k* by approximately 1.7 times (p < 0.001), lowering the *t*_*1/2*_ to 1.8 min. Increasing the w/w% of NIF to PVP had a negative effect on *k* (Fig. 7D) with 50 and 70% w/w NIF in PVP showing similar results to the nifedipine as received. Kaushal *et al*.^6^ described high drug content solid solutions containing small crystals within the dispersion, this was observed in >50% w/w FD NIF in PVP formulations of this study (Figs 3, 4 and 5). The formation of small crystals within solid dispersion limits the molecular dispersion of drug and polymer, and so reduces dissolution enhancement within amorphous formulations^6^. The type and content of polymer used in solid solutions has a great influence over the lowering of the contact angle of drug, a measure of wettability, where the limit of contact angle is 0°, indicating complete wetting, and 180° for no wetting^27^. PVP, with a contact angle of 27 ± 0.5° ^27^ was described to be much more efficient than other polymers (HPMC and Copovidone) in lowering contact angles of PWSD’s, such as crystalline cilostazol (68 ± 0.6°).

Measuring the nifedipine concentration in the dissolution vessel at 5 and 60 min gave the kinetic solubility. The kinetic solubility of the crystalline as received nifedipine was not affected by the presence of PVP in the dissolution media, (Figure 2S). Increasing the content of PVP in the amorphous freeze-dried formulations, significantly enhanced the kinetic solubility of nifedipine in the dissolution medium. This was demonstrated through the observed linear correlations; NIF_conc.5min_ = 19.3 × *C* + 11.04 and NIF_conc.60min_ = 19.6 × *C* + 11.96, where *C* is the % w/w NIF in PVP within freeze-dried formulations.

The thermodynamic solubility of nifedipine as received, determined over 5 days of equilibrium at (37.0 ± 0.5 °C), also showed a significant linear correlation (p < 0.05) with the concentration of PVP in dissolution medium. For these experiments the concentration range of PVP in the dissolution medium of 0–0.022% w/v (Figure 2S) was chosen to cover the range of PVP concentrations resulting from full dissolution of the 10—100% w/w NIF in PVP formulation within the 900 mL of dissolution medium. As presented in Figure 2S, a linear correlation between the concentration of PVP in the dissolution medium and the thermodynamic solubility of crystalline NIF was observed, following the equation [NIF_thermo-solubility_ = 134.62*X* +7.11] where *X* is the concentration of PVP in dissolution medium in % w/v units. However, the thermodynamic solubility of NIF in 0% w/v PVP in dissolution medium (0.1 M HCl pH = 1.2 and 37.0 ± 0.5 °C) was found to be lower than predicted by the above equation, equalling 6.8 ± 0.1 µg/mL at 37 ± 0.5 °C. Literature reference for the thermodynamic solubility of nifedipine in 0.1 M HCl was not available, however, solubility of nifedipine in buffer solutions with pH values ranging from 4–13 was published by Ali^31^ to fluctuate between 5.6–7.8 µg/mL^31, 59^. Thus our studies have revealed that the presence of PVP in the dissolution medium is not enough to increase the kinetic solubility or the dissolution rate of nifedipine. The polymer, PVP, must be in high concentrations in the vicinity of the drug to permit enhancement in dissolution rate. Molecularly dispersing the drug in large ratios of polymer furthers the enhancement of dissolution rate, as it increases the surface area between drug and polymer, and thus allow better wetting of the drug.

### Comparison of *in-situ* freeze-dried capsule with the liquid filled equivalents

**Figure 8:**
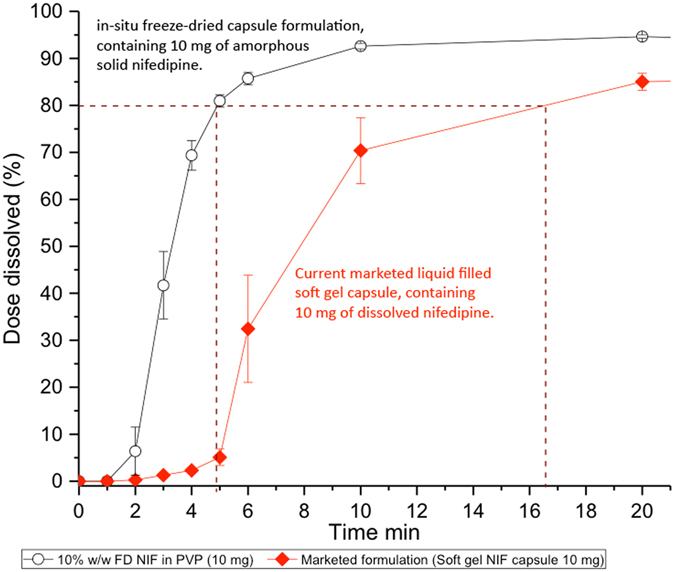
Comparing the dissolution profile of the *in-situ* capsule FD formulation 10% w/w NIF in PVP against the soft gel (liquid filled) marketed formulation TEVA 10 mg NIF. Average T80 for the marketed formulation is approximately 3 times longer than that of the *in-situ* capsule FD formulation (10% w/w NIF in PVP). Error bars represent standard error of n = 3.

The dissolution profile of the freeze-dried capsule containing 10% w/w NIF in PVP was compared to that of the soft gel, liquid filled, marketed TEVA formulation (Fig. 8), which consists of a lipid based solution of the drug in oil, sealed within a soft gelatin capsule^60, 61^. The freeze-dried capsule reached the BP recommended dissolution of 80% in less than 5 min, while the liquid filled capsule took more than 3 times longer to reach the same percentage of dose dissolved, (Fig. 8). The dissolution profile of the liquid filled capsule showed a lag period, whereby the slow dissolving soft capsule shell released the formulation at approximately 5 min^61^. In contrast, the equivalent *in-situ* freeze-dried capsules had a much reduced lag period of only 1 min, as a result of the capsule’s hard gelatin but fast dissolving composition (Fig. 8). Discounting the lag time from both dissolution profiles revealed that the *in-situ* freeze-dried formulation only required 4 min for 80% of the drug to dissolve in the aqueous medium. While the liquid based dosage form required an additional 11 min (approximately 3 times longer), after subtraction of the lag time for an equivalent 80% dissolution. Taking into account the difference in capsule performance, it is quite remarkable that a liquid formulation has slower dissolution than a solid amorphous formulation. The mechanism which underpins this difference is the time required for the hydrophobic nifedipine to diffuse from the oil/lipid droplets into the aqueous dissolution media^60, 62^ Such diffusional limitations are not an issue for the dissolution of PWSDs from the amorphous solid state especially when bound to polymers with a high affinity for water, as is the case when PVP is present^27^.

### Stability and utilisation of formulation platform

ICH derived stability study was performed on the freeze-dried capsules, 10% w/w NIF in PVP, at storage temperatures ranging from 25–37 °C and relative humidities ranging from 57–74% RH. The product was packaged in amber vials (Fig. 2), and sealed under nitrogen, conditions that replicate aluminium blister packaging used for commercial capsule formulations^63^. Quality control tests, in accordance to BP and ICH guidelines and regulations^64^, were performed on the product at time point 0, 1 and 3 months to test for the physical and chemical stability as well as product performance. The data confirms that the amorphous *in-situ* freeze-dried capsules were physically and chemically stable at elevated temperatures for 3 months (Table 1). Thus *in-situ* freeze-dried capsules may have the potential to remove the barriers associated with the formulation of amorphous drugs^6, 10, 27, 34, 65^.

**Table 1:** Stability study summary for the *in-situ* freeze-dried capsule formulation, 10% w/w NIF in PVP. All data = averages ± SE of n = 6.

Stability type	Parameters	Specification	Average measurements					
			0 months		3 months 25 °C 57% RH		3 months 37 °C 74% RH	
Physical	Weight of unit dosage form (mg)	201 ± 20	201.57	± 0.30	200.40	± 0.31	201.06	± 0.65
	*Tg* (°C) 2nd heat	—	130.26	± 3.56	125.04	± 2.19	125.16	± 0.76
	Crystalline NIF % w/w	0%	0.00	± 0.00	0.00	± 0.00	0.00	± 0.00
Chemical	Degradation products (%)*	<5%	0.00	± 0.00	0.00	± 0.00	0.00	± 0.00
Performance	Drug content (mg)	10 ± 0.5	9.61	± 0.12	9.37	± 0.10	9.44	± 0.06
	Rate constant for dissolution, k, (min^−1^)	—	0.37	± 0.05	—	—	0.39	± 0.04
	T80 (min)	<20 min	4.80	± 0.20	—	—	5.27	± 0.1

*Degradation products include: 4-(2-nitrophenyl) pyridine homologue and 4-(2-nitrosophenyl)-pyridine homologue.

## Conclusion

A novel *in-situ* freeze-dried capsule was successfully developed, and allowed the stable manufacture of amorphous solid formulations of the PWSD model, nifedipine. The use of TBA as an organic solvent was vital for the success of the novel *in-situ* freeze-drying method as it successfully dissolved both the drug and polymer without damaging the hard gelatine capsule shell. The inclusion of PVP in high w/w percentages increased the *T*_*g*_ and further stabilised amorphous nifedipine through hydrogen bond interactions between the C=O group of PVP and the N-H group of nifedipine. Maintaining nifedipine in its amorphous state greatly enhanced its dissolution rate and kinetic solubility. Dissolution studies showed the amorphous freeze-dried, 10% NIF in PVP, formulation to have the highest dissolution rate constant (0.37 ± 0.05 min^−1^) and lowest t_1/2_ (1.88 ± 0.05 min) of all the formulations, reaching T_80_ in less than 5 min, which is more than 3 times faster than the equivalent soft gel, liquid filled, marketed formulation. Furthermore, the *in-situ* capsule showed physical and chemical stability over 3 month accelerated storage at 37 °C and 75%RH and therefore meeting BP guidelines. The novel *in-situ* freeze-dried capsules are ideal for early clinical trial testing of oral, novel class II BCS drugs with relatively high potency (dosage of ≤50 mg). In order to apply the authors’ new technology, a decision tree is available indicating where and where not *in-situ* freeze-dried capsules are applicable (Figure 2S). The authors’ current and future work is focused on applying this decision tree to a range of different poorly soluble compounds and optimizing the freeze drying cycle, as we believe that *in-situ* freeze-dried capsules will become a general method for formulating class II BSC drug molecules.

## Materials and Methods

### *In-situ* freeze-drying of nifedipine within capsules

#### Preparation of feed solution

A 2% w/v nifedipine (Sigma-Aldrich Gillingham UK) in TBA (Sigma-Aldrich Gillingham UK) stock solution was prepared by dissolving pre weighed 200 mg of nifedipine in 10 mL of TBA. 200 mg of PVP K10 (Sigma-Aldrich Gillingham UK) was added to the readymade nifedipine solution and dissolved, creating a solution with a 1 to 1 NIF:PVP ratio (equivalent to freeze-dried 50% NIF in PVP). Feed solutions with varying NIF:PVP ratios were prepared using the same method while maintaining the concentration of nifedipine in all feed solutions constant at 2% w/v.

#### Sub ambient differential scanning calorimetry

Sub-ambient studies on freeze concentrate samples, of NIF and PVP in TBA, were performed over a temperature range of −30 to 40 °C, using a DSC Q20 (TA Instruments, New Castle, DE, USA) with a refrigerated cooling accessory (RCS). The DSC cell was purged with 50 cm^3^ min^−1^ dry nitrogen and the RCS was purged with 150 cm^3^ min^−1^ dry nitrogen. The DSC cell was calibrated following the instrument manufacturer’s guidelines. Experimental conditions followed an equilibration at 25 °C for 5 min, ramp to −30 °C (10 °C/min), followed by a ramp to 40 °C (10 °C/min) and a ramp to −30 °C (10 °C/min). Samples were analysed in aluminium hermetic pans. All experiments were repeated three times and an average primary glass transition temperature (*T*_*g*_ *’*) was recorded for freeze concentrates with high and low NIF:PVP ratios, allowing estimation of the collapse temperature (*T*_*c*_). The sample size used was approximately 5 mg, with the mass for each experiment recorded accurately on a six-figure balance, (Micro balance: Sartorius UK Ltd).

#### Capsule filling

Up to 30 red opaque gelatin capsules size 0 (distributed by The Alchemists Apothecary) were fitted, facing upwards, into a capsule holder board, designed for this study. Using a positive displacement pipette, designed for viscous and volatile samples (Gilson), 0.5 mL of feed solution was transferred into each capsule bottom and allowed to freeze. Capsules containing frozen feed solution, of the same NIF:PVP ratio, were transferred into amber freeze-drying vials. The vials were then labelled with the corresponding w/w % of NIF in PVP and stored in a −80 °C freezer (10–14 h).

#### Freeze-drying cycle

Vials containing the frozen solutions in gelatin capsules were transferred from −80 °C freezer onto a freeze-drying shelf, maintained at ≤−15 ^°^C, within the drying chamber of a bench top freeze dryer (Lyotrap freeze dryer; LTE Scientific Ltd). The drying chamber was immediately sealed and a vacuum was created using a connected vacuum pump (RV3 Edwards). The internal pressure of the drying chamber was continually reduced to eventually reach the minimum possible pressure, which in the absence of any samples is 0.03 mbar. The freeze-drying cycle lasted 5 days and once complete, the vacuum was broken by releasing nitrogen gas into the drying chamber and sealing the vials immediately.

For a secondary drying stage, sample vials were then unsealed and immediately transferred into a vacuum desiccator containing P_2_O_5_ at ambient temperature (18 °C to 25 °C) and a reduced pressure of 0.69 to 0.86 bar, using a facility vacuum system (BUSCH vacuum pumps). The secondary drying stage was maintained for 24 ± 1 h^19^. Once the secondary drying stage was complete, the vacuum was broken by releasing nitrogen gas into the desiccator and sealing vials under dry nitrogen immediately.

#### Recapping and storing freeze-dried capsules

Using a sealed nitrogen bag all vials were opened under nitrogen, and individual capsule bottoms were capped with the appropriate capsule tops. Fully capped capsules were placed back in vials which were re-sealed under nitrogen and stored at room temperature (18–25 °C).

### Differential scanning calorimetry on freeze-dried cake

Experimental conditions for freeze-dried cake followed an equilibration at 25 °C for 5 min, ramp to 200 °C (10 °C/min), followed by a ramp to 25 °C (10 °C/min) and a ramp to 200 °C (10 °C/min). Samples were analysed in aluminium pin-holed hermetic pans. All experiments were repeated three times. The sample size used was approximately 5 mg, with the mass for each experiment recorded accurately on a six-figure balance.

### Environmental scanning electron microscopy (SEM) of capsule shell

SEM was used to image freeze-dried capsule shell bottoms and un-treated capsule shell tops. Prior to imaging, capsule shells were gold coated in an Emitech K550 sputter coater for 2 min at 20 mA. Imaging was carried out under a FEI/Philips XL 30 scanning electron microscope (Eindhoven, The Netherlands). The operating conditions were: vacuum pressure 7.19–7.52E-4 Pa, HV 5 kV, a gaseous secondary detector and a typical magnification of ×15, ×100 and ×500. Images were captured and saved digitally.

### ATR FT-IR analysis

All FT-IR spectra were collected at room temperature (18–25 °C) using a PerkinElmer attenuated total reflectance (ATR) FT-IR spectrometer with a Dura-sample II-Diamond ATR. The resolution was set to 4 cm^−1^ single scan as it is sufficient for determining the present chemical bonds^66^. Absorbance spectra were recorded over the range 4000–400 cm^−1^. The software Spectrum (Perkin Elmer, Seer Green, UK) was used to interpret and apply base line correction for the reduction of background noise.

### Cross polarization optical microscopy

An optical microscope slide of each of the samples of interest was produced by carefully placing an amount of 1 to 2 mg of the sample onto the surface of a glass microscope slide which was then covered with a cover-slip. In the interest of spreading the powder into a thin layer, a small amount of pressure and motion was applied to the cover-slip. The slide was then placed under the optical microscope instrument (Leica Microsystems UK Ltd) which was fitted with an imaging camera (QiFastCam), a cross polariser and a red 1 (λ) compensator plate to assess birefringence. Magnified pictures, of 5 X, 25 X and 40 X, were generated at ambient temperature and pressure using the QCapture Pro 6.0 software. Representative scale bars were added to all images using previously calibrated in built scale bars using a graticule.

### Quality control testing

The following tests were also performed on the marketed fast release formulation (TEVA) containing 10 mg of NIF in a liquid form inside a soft capsule. The excipients of the liquid capsule filling were: glycerol, purified water, saccharin sodium, peppermint oil and macrogol 400 (prepared from polyethylene glycol otherwise referred to as PEG). Excipients of the soft capsule shell are: gelatin, glycerol (85%), titanium dioxide (E171) and sunset yellow (E110). In comparison the excipients of the hard gelatine capsule shell used in this study are: gelatin, 0.19% w/w E123 Amaranth, 0.76% w/w E124 Ponceau-4R and 0.66% w/w E171 Titanium dioxide and sorption water content of 13–16 w/w%. According to the manufacturers, Capsugel, the soft capsule shell is also thicker than the hard gelatin capsule^67^.

#### Uniformity of weight and appearance

All FD capsules were examined for damage or cracks on the outer shell. This was performed as part of the recapping step described above. Additionally 3 capsules per batch of each of the w/w % formulations were weighed individually.

#### Drug content

The content of NIF per capsule was determined for 3 capsules per batch of each of the w/w % formulations. The content of drug was determined using a HPLC method, adapted from a previous study^68^, with bupivacaine as an internal standard. The Agilent 1100 HPLC system series was used in combination with a Gemini-NX C18 revers phase column (particle size 5 µm, width of 4.60 mm and length of 150mm). The mobile phase consisted of 68% (v/v) HPLC grade methanol and 32% (v/v) 0.1 M ammonium acetate (adjusted to pH 5.8 using acetic acid). An isocratic mobile phase was set to a flow of 1.0 mL/min. The flow was not interrupted between samples of a given experiment. The column oven was set to 37 °C and the DAD unit was set to monitor absorbance at 238 nm to allow separation and detection of nifedipine and its photo degradation products: 4-(2-nitrophenyl) pyridine homologue and 4-(2-nitrosophenyl)-pyridine homologue^31, 69^. This was further validated using LC-Mass spec. Injection volume was set to 20 µL and the injection needle was programmed to wash with 100% HPLC grade methanol after each sample.

The HPLC method was validated following ICH guidelines for specificity, linearity & range, detection & quantification limits, accuracy and precision and robustness^42^.

### Dissolution testing

Dissolution testing method was performed using apparatus 2 (paddle apparatus), of which all parameters were calibrated and adjusted in accordance to Appendix XII B-dissolution of the BP guidelines (MHRA, 2014b). The dissolution medium used in this test is 900 mL of 0.1 M HCL (MHRA, 2014b). The dissolution medium also contained the internal standard (bupivacaine) at a concentration of 50 µg/mL. This ensured all withdrawn samples to contain a constant concentration of internal standard to facilitate drug quantification analysis using the HPLC method described above.

The dissolution instrument was assembled in accordance to the BP specification of appendix XII B (MHRA, 2014b). In addition to avoid sample contamination or interference from the equipment used, any plasticizers containing tubing was avoided. A stainless steel cannula was installed to which a 0.45 µm non-adsorptive millipore polyvinylidene fluoride syringe membrane filter was fitted. Finally a luer slip polypropylene syringe was fitted to the cannula via the filter to enable sample uptake. The dissolution test was performed following British Pharmacopeia (MHRA, 2014b) dissolution method for conventional capsules and tablets. Further, to supress light related degradation of NIF, the dissolution test was performed in a dark room where only low-actinic light (650–700 nm) was used. Light coming from the dissolution instrument was minimized but not completely eliminated as dissolution parameters were constantly monitored.

Prior to starting the *in-vitro* dissolution tests, a loose piece of non-reactive stainless steel, wire helix with a few turns, was securely attached to the test capsule, to act as a capsule sinker, as recommended by the BP guidelines (MHRA, 2014b). The capsule was then dropped into the dissolution vessel, at which time 0 min was marked. Samples were withdrawn on 1, 2, 3, 4, 5, 6, 10, 20, 45 and 60 minutes. At each time interval a sample of 5 mL was withdrawn and injected into amber HPLC glass vials. Once the dissolution testing was complete, the HPLC vials were analysed immediately using the above described HPLC assay.

Method of dissolution testing was optimised, to specifically account for the extreme hydrophobic properties of nifedipine^31^, following previously performed studies^70–74^. Plasticizer containing tubing and syringes where replaced by polypropylene products, as preliminary results showed variations (RSD > 2%) in NIF concentration of repeat sampling (n = 3). In addition, the usually fitted 0.45 µm cannula filters were found inefficient in filtering NIF particles as observed in dissolution testing of the TEVA soft gel capsules of NIF. Listed information from manufacturer detailed that the pore size value cited, of the cannula filters, was an average value, indicating that particles above 0.45 µm may pass through. As a result, a membrane non-adsorptive millipore polyvinylidene fluoride syringe filter was added to further enhance the filtration of nifedipine particles. Non-sink conditions were implemented in dissolution studies, by not adding additional or external surfactant agents such as sodium dodecyl sulfate (SDS) to enhance the solubility of NIF and therefore create a sink condition in dissolution medium^59^. Non-sink conditions in dissolution tests have enabled the kinetic solubility of amorphous NIF to be determined. In addition, as the solubility of drug is a factor that influences dissolution rate, as demonstrated by Noyes and Whitney^55^, tests with non-sink conditions, unlike sink conditions, allow better discrimination between dissolution profiles of different formulations where the kinetic solubility of drug is predicted to be different^75^.

A multivariate approach (MANOVA) test^76, 77^ was conducted to investigate differences in dissolution profiles of different formulations of NIF (Fig. 7). Whereby dissolved % of the average dose, presented in Figure 1S, at individual time intervals are compared for any statistically significant differences. Alternatively, the non-parametric test Kruskal-Wallis, which assumes monotonic relation-ship between the two variables, was applied. Rate constants (*k*), and the related half-life (*t*_*1/2*_), of NIF dissolution for the different formulations (Fig. 7), determined by fitting dissolution profiles to the most representative kinetic model^77^, were also compared for any significant differences using student T-test^77–79^.

### Stability testing

#### Effect of TBA on the stability of gelatine capsule shells

A volume of 0.5 mL of liquid TBA (35 ± 1 °C) was added to the inside of a hard red gelatin capsule maintained inside a temperature regulated vial, using a temperature controlled water bath. TBA was maintained liquid inside the gelatin capsule for 60 min. Following this, the liquid TBA was pipetted out of the gelatin capsule shells and analysed for food colouring additives described as ingredients of the gelatin capsule shells by the manufacturers. The HPLC method used was described by^54^, and it used an isocratic mobile phase (20% v/v methanol, 80% v/v 0.1 M ammonium acetate at pH 5.8) at a flow rate of 1 mLmin^−1^. Absorbance at 510 nm was monitored for all samples.

#### Stability of FD *in-situ* capsule formulation

A stability study was performed by storing *in-situ* capsule FD formulations in nitrogen sealed amber vials under two conditions; The first condition 25 ± 2 °C and 57 ± 0.4% RH, was achieved by storage at a 25 ± 2 °C controlled room temperature, where by the samples were stored in a sealed box containing saturated solutions of sodium bromide salt^80^. The second condition 37 ± 2 °C and 74 ± 0.13% RH was achieved using the same method while a saturated sodium chloride salt solution was used to maintain the % RH^80^. Both conditions were monitored periodically to ensure temperature and humidity values are within in specified limits. Samples were withdrawn and tested for physical stability: % w/w of crystalline nifedipine, chemical stability: presence of chemical degradation products, and performance: unit dosage weight uniformity, drug content and dissolution profile.

## Electronic supplementary material

Supplementary Info
