# Supplementary material for: In-situ freeze-drying - forming amorphous solids directly within capsules: An investigation of dissolution enhancement for a poorly soluble drug
Source: Sci Rep. 2017 Jun 6;7:2910. doi: 10.1038/s41598-017-02676-2 (PMC5460206; doi:10.1038/s41598-017-02676-2)
Supplement: Supplementary file 1 — Supplementary Info [file 41598_2017_2676_MOESM1_ESM.pdf]

In-situ freeze-drying - forming amorphous solids directly within capsules: An investigation of dissolution enhancement for a poorly soluble drug.

Supplementary Information

Abdulmalik Alqurshi [a,b], K. L. Andrew. Chan [a], Paul G. Royall [a*]

[a] King's College London, Institute of Pharmaceutical Science, Franklin-Wilkins Building, 150 Stamford Street, London, UK, SE1 9NH, [b] Current address: Taibah University, College of Pharmacy, Prince Naif Bin Abdulaziz, Medina, Saudi Arabia

*Corresponding author & corresponding author contact details: e-mail: paul.royall@kcl.ac.uk; Telephone number: 020 7848 4369; Fax number: 020 7848 4500

KEYWORDS: nifedipine, PVP, amorphous, freeze-drying, capsules, dissolution rate enhancement, thermal analysis, light microscopy, FTIR.

Table 1S Uniformity of weight for unit dosage (n=3). All formulations are designed to contain 10 mg NIF. The percentage listed under formulation column details the w/w % of NIF in PVP of the *in-situ* capsule FD formulations. Expected weight of formulation is comprised of the average weight of empty capsule (90 mg ± 0.2%), the target weight of NIF (10 ± 0.5 mg) and the target weight of PVP depending on the NIF:PVP ratio used.

.

Formulation and % w/w of NIF in PVP	Expected weight of formulation (mg)	Average observed weight of unit dosage form (mg)	%RSD
Marketed	-	616.39	0.2%
FD 100%	100.00	100.37	4.4%
FD 70%	104.29	103.95	3.0%
FD 50%	110.00	109.72	2.3%
FD 30%	123.30	125.41	3.2%
FD 10%	190.00	201.57	1.4%

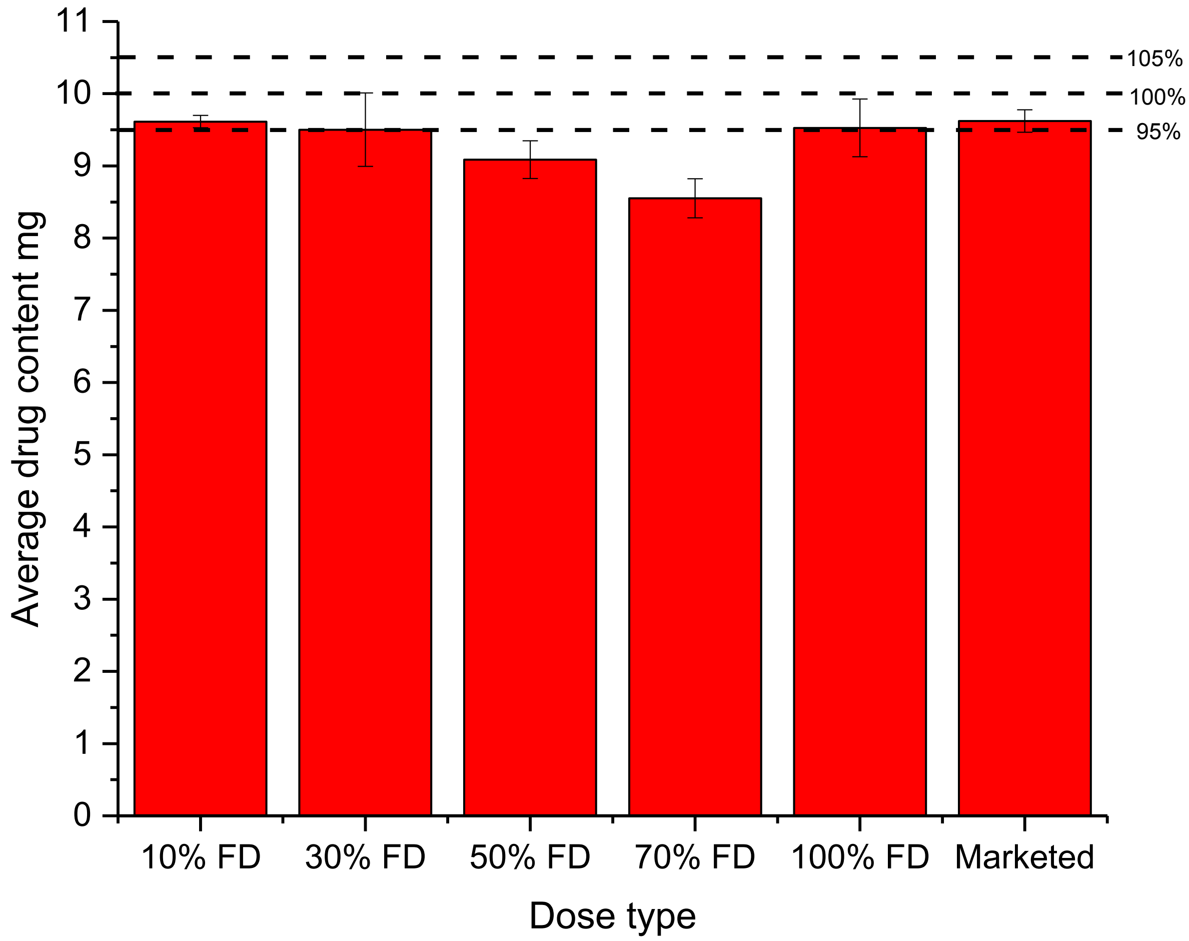

Figure 1S Average drug content in each of the tested formulations expressed in % w/w NIF in PVP as well as the soft gel (liquid filled) marketed formulation (TEVA 10 mg nifedipine soft capsule). Error bars represent standard error of n=3. The marked dashed lines indicate the BP drug content limits [81].

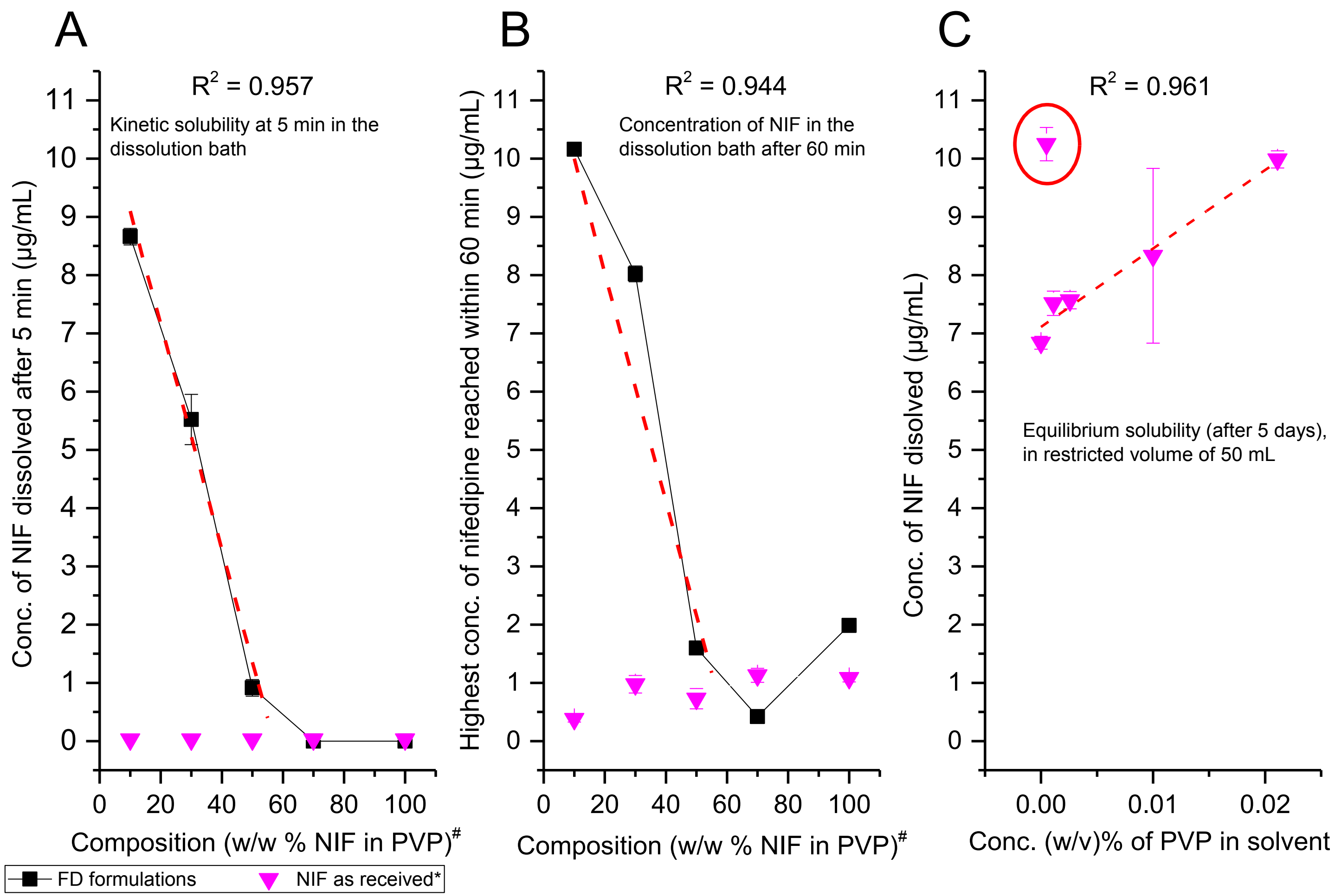

* Dissolution medium contains PVP at concentrations equavelent to the amount of PVP in corresponding FD formulations

Figure 2S Comparing the concentration of nifedipine **(A)** after 5 min of dissolution **(B)** after 60 min of dissolution and **(C)** after 5 days of equilibrium (thermodynamic solubility). The dissolution medium is 0.1M HCL, with the exception to NIF as received* in (A) & (B) Only when testing nifedipine as received*, the dissolution medium also contained PVP at concentrations equal to those resulted by the full dissolution of PVP from equivalent FD formulations in 900 mL 0.1M HCl (e.g. NIF as received* with an x-axis (composition w/w % NIF in PVP) of 50%, indicates that the dissolution medium for this particular test contained a pre-dissolved 10 mg of PVP). Error bars represent standard error (n=3). Circled data point in (C) was considered an outlier caused by an artefact, and therefore was not included in linear regression analysis.

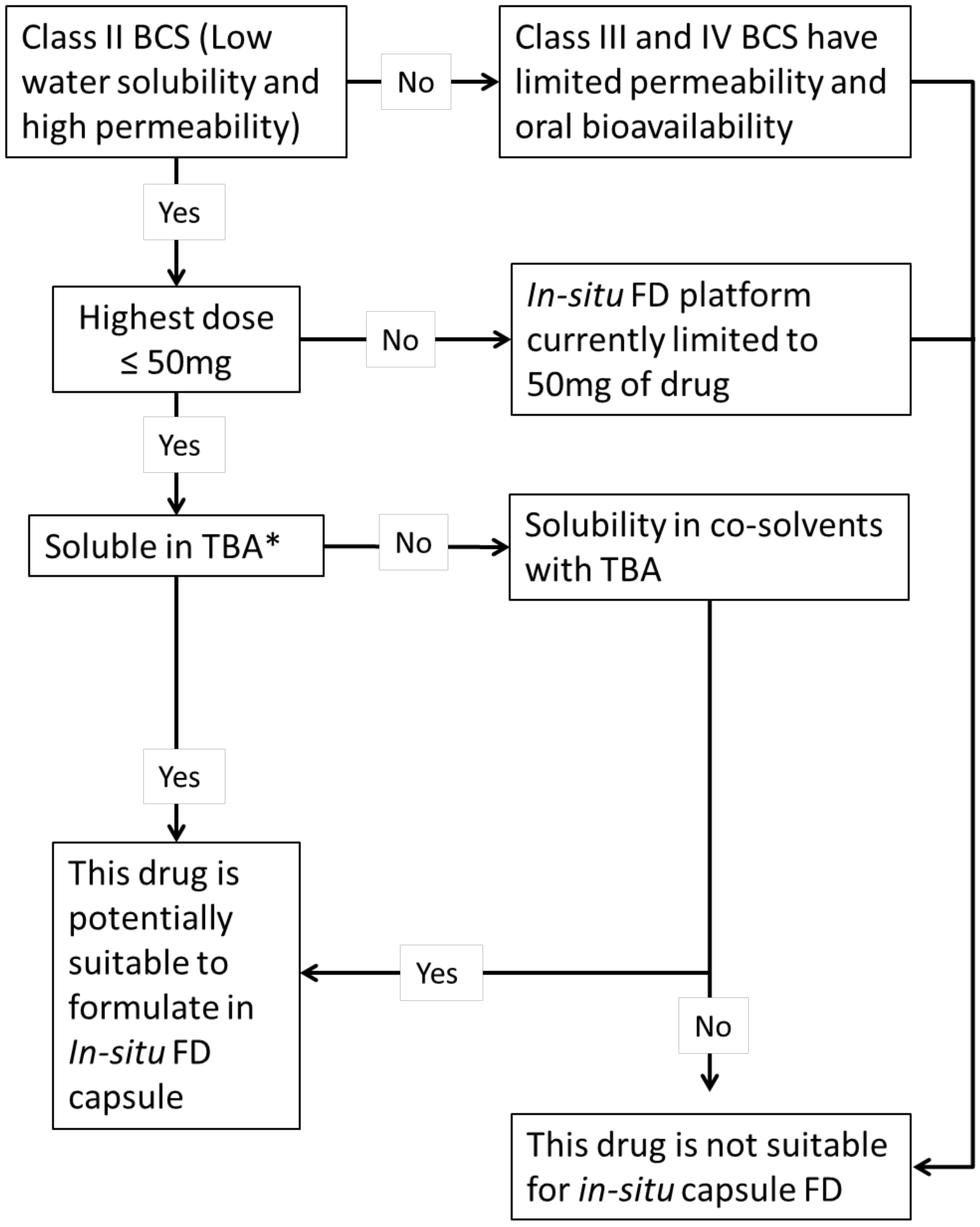

Figure 3S Flow diagram to help with evaluating potential drugs for formulating into the *in-situ* FD capsule platform to enhance dissolution rate. *Recommended dose of drug must be soluble in 0.5 mL of TBA.

Table 2S LOD and LOQ of crystalline nifedipine in PVP (% w/w).

Parameter	Source or equation	Value
Slope (S)	$\Delta_{melt}H = \left(1.01 \times \frac{w}{w} \% \; NIF\right) - 8.06$	1.01
Standard error of intercept (SE)	Regression analysis	1.57
LOD of crystalline NIF in w/w %	$= 3.3 \times \frac{\sigma}{S}$	5.15%
LOQ of crystalline NIF in enthalpy	$= 10 \times \frac{\sigma}{S}$	15.62%

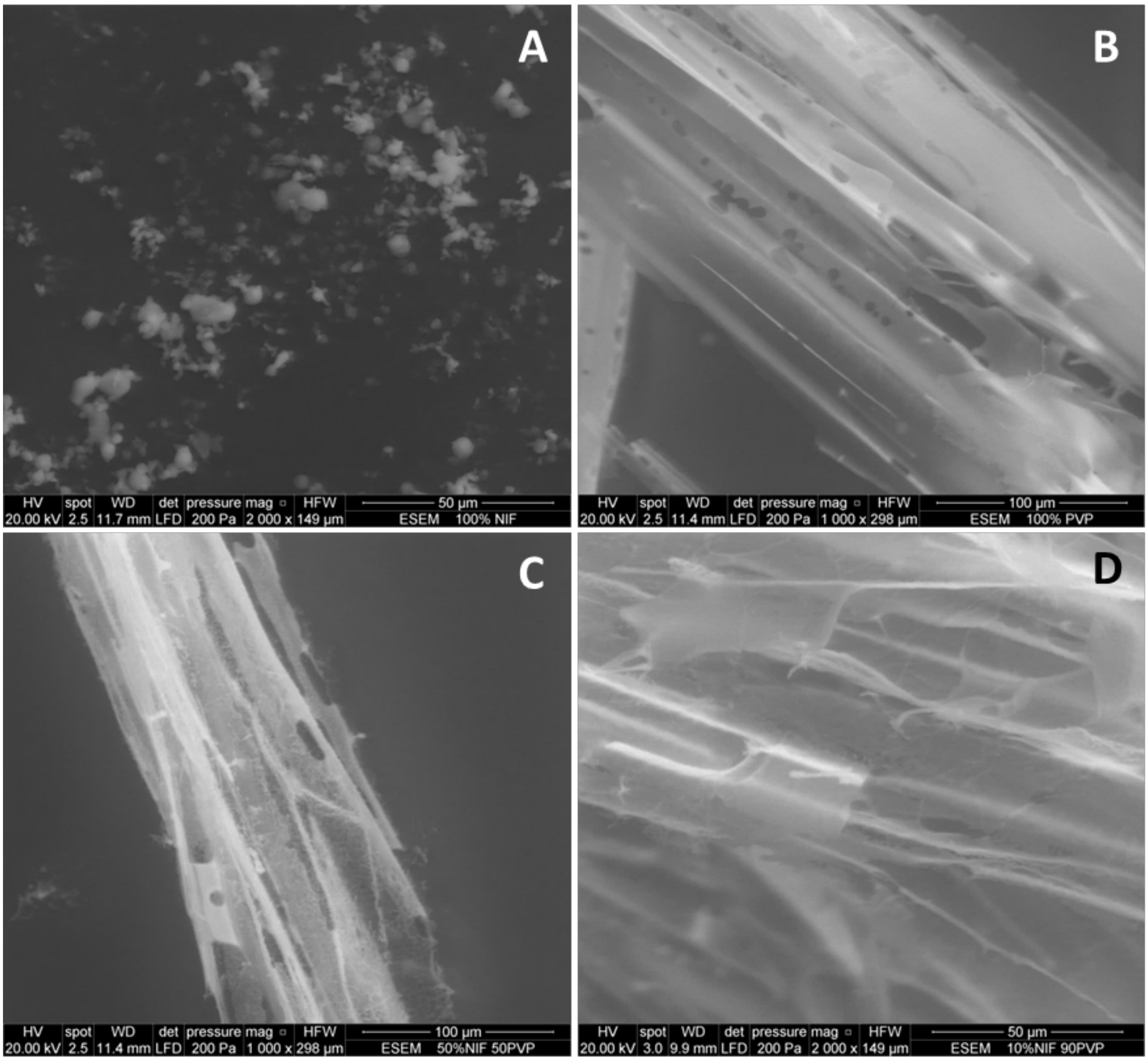

Figure 4S ESEM of **A)** freeze-dried 100% w/w NIF, **B)** freeze-dried 100% w/w PVP, **C)** & **D)** freeze-dried 50% w/w NIF in PVP and freeze dried 10% w/w NIF in PVP respectively.
